# Comparative Post-Transplant Outcomes in Alcohol-Related Liver Disease and Non-Alcohol Steatohepatitis: A Multicenter Propensity-Matched Study

**DOI:** 10.3390/medsci14020286

**Published:** 2026-06-01

**Authors:** Sajjad Ahmed Khan, Arkadeep Dhali, Hareesha Rishab Bharadwaj, Ashish Sharma, Saqr Alsakarneh, Islam Mohamed, Abdullah Sultany, Sahib Singh, Hassam Ali, Dushyant Singh Dahiya

**Affiliations:** 1 Department of Internal Medicine, Birat Medical College Teaching Hospital, Morang 56613, Nepal; 2 Department of Gastroenterology, Queen’s University Belfast, Belfast BT7 1NN, UK; 3 Department of Internal Medicine, Royal Stoke University Hospital, University Hospitals of North Midlands NHS, Stoke-on-Trent ST4 6QG, UK; 4 Department of Internal Medicine, Yale New Haven Hospital, New Haven, CT 06510, USA; 5 Department of Gastroenterology and Hepatology, Mayo Clinic Rochester, Rochester, MN 55905, USA; 6 Department of Gastroenterology and Hepatology, University of Missouri, Columbia, MO 65211, USA; 7 Department of Internal Medicine, Guthrie Robert Packer Hospital, Sayre, PA 18840, USA; abdullahsultany142160@gmail.com; 8 Department of Gastroenterology and Hepatology, SUNY Upstate Medical Center, Syracuse, NY 13210, USA; 9 Department of Gastroenterology, Hepatology and Nutrition, Brody School of Medicine, East Carolina University, Greenville, NC 27858, USA; 10 Division of Gastroenterology, Hepatology & Motility, The University of Kansas School of Medicine, Kansas City, KS 66160, USA

**Keywords:** liver transplantation, alcohol-related liver disease, non-alcoholic steatohepatitis, outcomes

## Abstract

**Background:** Liver transplantation (LT) remains the definitive treatment for end-stage liver disease; however, post-LT outcomes may differ depending on underlying disease etiology. Our study aimed to compare post-LT outcomes between alcoholic and non-alcoholic steatohepatitis (NASH)-related liver transplant recipients in the United States. **Methods:** A retrospective cohort study was conducted using the TriNetX Research Network. Adult liver transplant recipients (≥18 years) were categorized into two mutually exclusive cohorts: alcoholic liver disease and NASH-related liver disease. Propensity score matching (1:1) was performed to balance baseline characteristics. Clinical outcomes were assessed. Comparative analyses included risk ratios, risk differences, odds ratios, Kaplan–Meier survival analysis, and hazard ratios with log-rank testing. **Results:** Compared with NASH recipients, alcoholic LT recipients had a significantly higher risk of rejection (14.5% vs. 12.1%; RR 1.195, 95% CI 1.076–1.327; *p* = 0.001) and hepatic encephalopathy (14.3% vs. 8.3%; RR 1.720, 95% CI 1.526–1.938; *p* < 0.001). Acute kidney injury was also more frequent in the alcoholic cohort (47.3% vs. 43.6%; RR 1.084, 95% CI 1.036–1.133; *p* < 0.001). In contrast, sepsis (15.6% vs. 18.0%; RR 0.869, 95% CI 0.794–0.952; *p* = 0.003) and CKD (46.1% vs. 51.1%; RR 0.901, 95% CI 0.863–0.939; *p* < 0.001) occurred less frequently in alcoholic LT patients. No significant differences were observed for liver transplant failure or ascites. Kaplan–Meier analyses demonstrated significantly lower rejection-free survival (HR 1.241, *p* < 0.001), higher hepatic encephalopathy (HR 1.808, *p* < 0.001), increased AKI risk (HR 1.150, *p* < 0.001) and higher all-cause mortality in the alcoholic cohort (HR 1.106, *p* = 0.031). **Conclusions:** In this large real-world matched cohort study, alcoholic LT recipients demonstrated higher risks of complications and all-cause mortality compared with NASH LT recipients.

## 1. Introduction

Liver transplantation is the definitive therapeutic intervention for patients with end-stage liver disease, offering substantial improvement in survival and quality of life across a broad spectrum of hepatic pathologies [1]. Despite advances in surgical techniques, perioperative care, and immunosuppressive regimens, post-transplant outcomes remain heterogeneous and are strongly influenced by the underlying etiology of liver disease [2]. Understanding etiological differences in post-transplant morbidity and mortality is essential for optimizing recipient selection, tailoring follow-up strategies, and improving long-term graft and patient survival [3].

Alcohol-related liver disease (ALD) and non-alcoholic steatohepatitis (NASH) represent two of the most common indications for liver transplantation worldwide [4]. ALD continues to be a major contributor to liver failure in many regions, often associated with complex psychosocial determinants, relapse risk, and multisystem comorbidities [5]. In contrast, NASH has emerged as a rapidly growing indication for transplantation, driven by the global epidemics of obesity, type 2 diabetes mellitus, and metabolic syndrome [6]. Unlike ALD, NASH-related liver disease is typically associated with a high burden of cardiometabolic risk factors, including hypertension, dyslipidemia, and chronic kidney disease, which may independently influence post-transplant outcomes [7].

Although both conditions ultimately lead to cirrhosis and liver failure requiring transplantation, their systemic profiles differ significantly. ALD patients often present with complications related to chronic alcohol exposure, malnutrition, and neuropsychiatric comorbidities, while NASH patients typically exhibit features of metabolic dysfunction and cardiovascular disease [8]. These differences raise important questions regarding comparative post-transplant outcomes, including graft rejection, infection risk, renal dysfunction, and overall survival. However, existing evidence remains inconsistent. Large-scale real-world data analyses offer an opportunity to overcome these limitations by evaluating outcomes across diverse populations and healthcare settings. The use of federated electronic health record networks, such as TriNetX, enables robust comparative effectiveness research while maintaining patient privacy [9]. Such platforms also facilitate propensity score matching, allowing more accurate adjustment for baseline differences between cohorts and improving the validity of comparative analyses.

In this context, the present study aimed to compare post-liver transplantation outcomes between patients with alcoholic liver disease and those with NASH using a large multicenter electronic health record database. We specifically evaluated clinically relevant outcomes, including liver transplant rejection, graft failure, infectious complications, renal dysfunction, and all-cause mortality. We hypothesized that ALD would be associated with a distinct pattern of immunological and neuropsychiatric complications, whereas metabolic comorbidities would dominate in NASH. A clearer understanding of these differences may have important implications for post-transplant monitoring strategies, immunosuppressive management, and long-term patient counseling. Furthermore, identifying high-risk subgroups may support the development of targeted interventions aimed at reducing preventable complications and improving graft longevity.

## 2. Methodology

### 2.1. Study Design and Data Source

This retrospective, multicenter cohort study utilized de-identified electronic health record data from the TriNetX Research Network [© TriNetX, LLC], which aggregates longitudinal clinical information from 112 healthcare organizations. The federated analytics approach enabled analysis across sites without patient-level data sharing, ensuring data privacy while supporting large-scale real-world evidence generation.

The index event was defined as the earliest recorded liver transplant status (ICD-10-CM Z94.4) combined with a disease-specific diagnosis code. This approach was chosen to enhance cohort capture given variability in procedural coding completeness across institutions and potential inconsistency in recording liver transplant procedure codes. We acknowledge that transplant status codes may not coincide with the actual transplantation event, introducing potential variability in lead-in time and risk of misclassification of time zero. Although immortal time bias was partially mitigated by excluding outcomes occurring on the index day, residual bias related to delayed coding may persist.

### 2.2. Study Population

The study population consisted of adult patients (aged ≥ 18 years) who had undergone liver transplantation. Two mutually exclusive cohorts were defined based on underlying liver disease etiology at the time of transplantation.

The Alcoholic Liver Transplant cohort included patients with documented liver transplant status (ICD-10-CM: Z94.4) in combination with alcoholic liver disease etiologies, including alcoholic cirrhosis (with or without ascites), alcoholic hepatic failure without coma, or unspecified alcoholic liver disease. Patients with coexisting diagnoses of viral hepatitis, autoimmune hepatitis, non-alcoholic fatty liver disease, non-alcoholic steatohepatitis, primary biliary cirrhosis, cholangitis, or hepatic malignancy were excluded to ensure diagnostic specificity.

The NASH Liver Transplant cohort included patients with liver transplant status (ICD-10-CM: Z94.4) and a documented diagnosis of non-alcoholic steatohepatitis or fatty liver disease. Patients with alcoholic liver disease, viral hepatitis, autoimmune hepatitis, cholestatic liver disease, or hepatic malignancy were excluded.

### 2.3. Index Event Definition and Follow-Up

The index event was defined as the earliest recorded occurrence of liver transplant status combined with the respective underlying liver disease diagnosis. The date of the index event was considered time zero for follow-up.

Outcomes were assessed beginning one day after the index event to avoid immortal time bias. No fixed upper limit for follow-up was imposed; patients were followed until the last available clinical record within the database. Patients with index events occurring more than 20 years prior to analysis were excluded.

Follow-up duration was inherently variable across individuals, reflecting real-world utilization of longitudinal electronic health records. No fixed administrative censoring time was imposed. Consequently, Kaplan–Meier estimates represent cumulative event-free survival probabilities over heterogeneous observation periods, rather than survival probabilities at standardized time points (e.g., 1-year or 5-year survival). This variability in follow-up time should be considered when interpreting end-of-observation survival estimates.

### 2.4. Outcomes

Outcome definitions were based on ICD-10-CM diagnostic codes extracted from the TriNetX electronic health record network. Sepsis was defined using ICD-10-CM code A41, which captures clinically diagnosed sepsis but does not allow application of Sepsis-3 criteria, which require standardized assessment of organ dysfunction and clinical parameters. CMV disease was identified using ICD-10-CM code B25; however, it is important to note that this does not distinguish between asymptomatic CMV infection, CMV syndrome, or tissue-invasive CMV disease as defined by the international consensus criteria, and therefore likely reflects only clinically coded CMV disease episodes rather than full virologically stratified CMV status. Hepatic encephalopathy (K76.82), ascites (R18), acute kidney injury (N17), chronic kidney disease (N18), and liver transplant rejection (T86.41) were similarly identified using validated administrative codes; however, these do not provide clinical grading or severity stratification and may not fully capture subclinical or biopsy-proven disease. In particular, liver transplant rejection codes do not differentiate between acute cellular, antibody-mediated, or chronic rejection, while graft failure (T86.42) represents a heterogeneous composite outcome including re-transplantation and death. Importantly, key transplant-specific clinical variables, including donor and recipient CMV serostatus, CMV prophylaxis or pre-emptive therapy strategies, immunosuppressive regimens, biopsy findings, laboratory parameters, and severity scores (e.g., MELD), were not available within the database. Consequently, CMV-related outcome interpretation could not be stratified by pre-transplant serostatus or antiviral intervention exposure, and this remains a significant limitation. Finally, while the present study focuses on clinical outcomes, the associated healthcare resource utilization and economic burden particularly related to complications such as CMV disease, rejection, sepsis, and renal dysfunction were not directly assessed in this analysis but represent an important area for future research given their potential implications for post-transplant cost and healthcare system burden.

### 2.5. Statistical Analysis

A propensity score matching approach was applied to minimize baseline confounding between cohorts. Matching was performed at a 1:1 ratio, resulting in 4601 patients in each cohort. Covariates included demographic characteristics (age, sex, race, ethnicity), comorbid conditions, and baseline medication exposures. Matching adequacy was assessed using standardized mean differences, with values < 0.1 considered acceptable balance.

After matching, comparative analyses were conducted using risk-based measures and time-to-event methods. Risk differences, risk ratios, and odds ratios were calculated for each outcome, with corresponding 95% confidence intervals. Kaplan–Meier survival analyses were used to evaluate cumulative event-free survival, and group comparisons were performed using the log-rank test. Hazard ratios were estimated using proportional hazards models. A two-sided *p*-value of <0.05 was considered statistically significant.

### 2.6. Data Handling and Software

All analyses were performed using the TriNetX Analytics platform, which provides real-time federated querying across participating healthcare organizations. Data are de-identified in accordance with the Health Insurance Portability and Accountability Act (HIPAA) standards, and no patient-level data were exported from the network.

### 2.7. Ethical Considerations

The study utilized de-identified secondary data; therefore, informed consent was not required. The TriNetX platform complies with relevant institutional and regulatory requirements for data privacy and research ethics. The study was conducted in accordance with the Declaration of Helsinki principles.

## 3. Results

### 3.1. Study Population and Propensity Score Matching

A total of 13,348 liver transplant recipients were identified from the TriNetX Research Network (112 healthcare organizations), including 6877 patients in the Alcoholic Liver Transplant cohort and 6471 patients in the NASH Liver Transplant cohort (Table 1). After 1:1 propensity score matching, 4601 patients remained in each group and were included in the final comparative analysis (Table 2).

Baseline characteristics were well balanced after matching. The mean age was similar between the Alcoholic and NASH cohorts (55.0 ± 10.7 years vs. 55.3 ± 13.9 years, respectively). The proportion of female patients was 37.4% in the Alcoholic cohort and 36.5% in the NASH cohort. Standardized mean differences for most covariates were <0.05, indicating adequate balance across demographic variables, comorbidities, and medication exposures.

### 3.2. Liver Transplant Rejection

Liver transplant rejection occurred more frequently in the Alcoholic Liver Transplant cohort compared with the NASH cohort. The cumulative incidence was 14.5% (668/4601) in the Alcoholic group and 12.1% (559/4601) in the NASH group. This corresponded to a risk difference of 0.024 (95% CI 0.010–0.038, *p* = 0.001) and a risk ratio of 1.195 (95% CI 1.076–1.327), indicating a statistically significant increased risk in the Alcoholic cohort.

**Figure 1:**
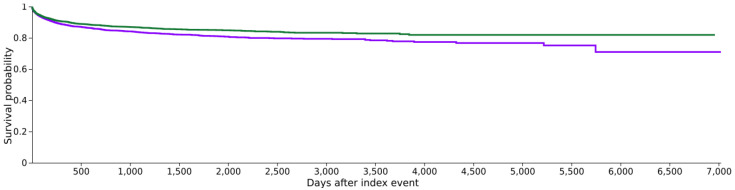
Kaplan–Meier curve for liver transplant rejection. Purple: Alcohol-related liver disease cohort. Green: Non-alcoholic steatohepatitis cohort.

Time-to-event analysis confirmed worse rejection-free survival in the Alcoholic cohort, with a log-rank test χ^2^ of 14.27 (*p* < 0.001) (Figure 1). The hazard ratio was 1.241 (95% CI 1.109–1.389), indicating a 24% higher hazard of rejection compared with the NASH cohort. At the end of follow-up, survival probability free from rejection was 70.94% in the Alcoholic cohort compared with 81.87% in the NASH cohort.

### 3.3. Liver Transplant Failure

No statistically significant difference was observed in liver transplant failure between the two cohorts. The incidence was 7.8% (360/4601) in the Alcoholic group and 7.4% (341/4601) in the NASH group. The risk difference was 0.004 (95% CI −0.007 to 0.015, *p* = 0.455), and the risk ratio was 1.056 (95% CI 0.916–1.217), indicating no meaningful between-group difference.

**Figure 2:**
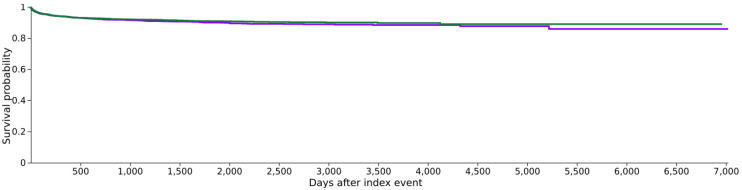
Kaplan–Meier curve for liver transplant failure. Purple: Alcohol-related liver disease cohort. Green: Non-alcoholic steatohepatitis cohort.

Kaplan–Meier analysis similarly showed no significant difference in transplant failure-free survival (log-rank *p* = 0.294) (Figure 2). The hazard ratio was 1.082 (95% CI 0.933–1.255), and survival at end of follow-up was 85.87% in the Alcoholic cohort and 88.97% in the NASH cohort.

### 3.4. Ascites

Ascites was a common post-transplant outcome in both cohorts, with similar incidence rates observed. It occurred in 33.7% (1549/4601) of the Alcoholic cohort and 32.6% (1502/4601) of the NASH cohort. The risk difference was 0.010 (95% CI −0.009 to 0.029, *p* = 0.298), and the risk ratio was 1.031 (95% CI 0.973–1.093), indicating no statistically significant difference.

Although Kaplan–Meier analysis suggested a borderline difference in survival curves (log-rank *p* = 0.100), the hazard ratio was 1.061 (95% CI 0.988–1.139), which did not reach statistical significance for proportional hazards at the conventional threshold.

### 3.5. Hepatic Encephalopathy

Hepatic encephalopathy occurred significantly more frequently in the Alcoholic Liver Transplant cohort. The incidence was 14.3% (657/4601) in the Alcoholic group compared with 8.3% (382/4601) in the NASH group. This corresponded to a risk difference of 0.060 (95% CI 0.047–0.073, *p* < 0.001) and a risk ratio of 1.720 (95% CI 1.526–1.938), indicating a substantially higher risk in the Alcoholic cohort.

Kaplan–Meier analysis confirmed significantly worse encephalopathy-free survival in the Alcoholic cohort (log-rank χ^2^ = 87.28, *p* < 0.001), with a hazard ratio of 1.808 (95% CI 1.594–2.051).

### 3.6. CMV Disease

Cytomegalovirus disease showed no statistically significant difference in overall risk between cohorts. The incidence was 13.6% (624/4601) in the Alcoholic cohort and 12.5% (576/4601) in the NASH cohort. The risk difference was 0.010 (95% CI −0.003 to 0.024, *p* = 0.137), and the risk ratio was 1.083 (95% CI 0.975–1.204).

However, Kaplan–Meier analysis demonstrated a statistically significant difference in time-to-event distribution (log-rank *p* = 0.040), with a hazard ratio of 1.126 (95% CI 1.005–1.261), suggesting a modestly increased hazard in the Alcoholic cohort.

### 3.7. Sepsis

Sepsis occurred significantly less frequently in the Alcoholic cohort compared with the NASH cohort. The incidence was 15.6% (719/4601) in the Alcoholic group and 18.0% (827/4601) in the NASH group. This corresponded to a risk difference of −0.023 (95% CI −0.039 to −0.008, *p* = 0.003) and a risk ratio of 0.869 (95% CI 0.794–0.952), indicating a lower risk among Alcoholic transplant recipients.

Kaplan–Meier analysis supported this finding (log-rank *p* = 0.027), with a hazard ratio of 0.893 (95% CI 0.808–0.987), suggesting a protective association in the Alcoholic cohort. However, the absolute difference in sepsis incidence between the cohorts was modest, and the lower bound of the hazard ratio approached unity, warranting cautious interpretation of this association. In addition, unmeasured variables such as obesity severity, glycemic control, nutritional status, and post-transplant immunosuppression intensity may have contributed to residual confounding. Therefore, the observed lower sepsis risk in the Alcoholic cohort should not be interpreted as definitively protective but rather as a hypothesis-generating finding requiring further prospective validation.

### 3.8. Acute Kidney Injury

Acute kidney injury occurred more frequently in the Alcoholic cohort. The incidence was 47.3% (2175/4601) compared with 43.6% (2007/4601) in the NASH cohort. This corresponded to a risk difference of 0.037 (95% CI 0.016–0.057, *p* < 0.001) and a risk ratio of 1.084 (95% CI 1.036–1.133). Kaplan–Meier analysis demonstrated significantly worse AKI-free survival in the Alcoholic cohort (log-rank *p* < 0.001), with a hazard ratio of 1.150 (95% CI 1.082–1.221).

### 3.9. Chronic Kidney Disease

Chronic kidney disease was significantly less frequent in the Alcoholic cohort compared with the NASH cohort. The incidence was 46.1% (2119/4601) in the Alcoholic group and 51.1% (2353/4601) in the NASH group. This corresponded to a risk difference of −0.051 (95% CI −0.071 to −0.030, *p* < 0.001) and a risk ratio of 0.901 (95% CI 0.863–0.939). Kaplan–Meier analysis confirmed a significant difference in CKD-free survival (log-rank *p* < 0.001), with a hazard ratio of 0.879 (95% CI 0.829–0.932).

### 3.10. All-Cause Mortality

**Figure 3:**
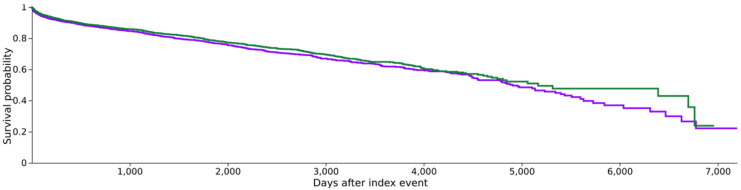
Kaplan–Meier curve forAll-Cause Mortality. Purple: Alcohol-related liver disease cohort. Green: Non-alcoholic steatohepatitis cohort.

All-cause mortality was significantly higher in the Alcoholic Liver Transplant cohort. During follow-up, 939 deaths occurred in the Alcoholic group compared with 878 in the NASH group. Kaplan–Meier analysis showed a statistically significant difference in survival (log-rank *p* = 0.031), with a hazard ratio of 1.106 (95% CI 1.009–1.213), indicating a higher risk of death in the Alcoholic cohort (Figure 3). Survival probability at the end of follow-up was 22.17% in the Alcoholic cohort compared with 23.85% in the NASH cohort.

## 4. Discussion

This large multicenter real-world cohort study compared post-liver-transplantation outcomes between recipients with alcoholic liver disease and those with non-alcoholic steatohepatitis (NASH) using a propensity score-matched analysis. The findings demonstrate clinically meaningful differences in several post-transplant complications despite overall similarity in many graft-related outcomes. Specifically, alcoholic liver transplant recipients exhibited higher risks of liver transplant rejection, hepatic encephalopathy, acute kidney injury (AKI), and all-cause mortality, while NASH recipients showed a higher burden of chronic kidney disease (CKD) and sepsis. No significant differences were observed in transplant failure or ascites. These results highlight that underlying disease etiology continues to influence post-transplant morbidity patterns even after adjustment for baseline demographic and clinical confounders [10].

One of the most important findings of this study is the significantly increased risk of liver transplant rejection among alcoholic liver disease recipients. The observed higher hazard of rejection in the alcoholic cohort may reflect several biological and behavioral mechanisms. Alcohol-related liver disease is often associated with immune dysregulation, malnutrition, and systemic inflammation, all of which may contribute to altered immune responses following transplantation [11]. In addition, variability in adherence to immunosuppressive therapy, which has been previously reported in populations with alcohol use disorders, may further increase rejection risk. These findings suggest that this subgroup may require closer immunological monitoring and reinforced adherence strategies in the post-transplant period.

Hepatic encephalopathy was also significantly more common in the alcoholic cohort. This may be explained by pre-existing neurocognitive vulnerability associated with chronic alcohol exposure, as well as potential differences in post-transplant metabolic recovery [12]. Alcohol-related neurotoxicity, micronutrient deficiencies (particularly thiamine), and persistent cognitive impairment may predispose these patients to recurrent or persistent encephalopathic episodes even after liver replacement [13]. The magnitude of difference observed in this study indicates that hepatic encephalopathy remains a substantial post-transplant complication in this population and may warrant targeted neuropsychiatric follow-up and early intervention strategies [14].

In contrast, NASH recipients demonstrated a higher burden of chronic kidney disease. This finding is consistent with the established association between metabolic syndrome, diabetes mellitus, and progressive renal dysfunction [15]. NASH is fundamentally a systemic metabolic disease, and patients frequently present with coexisting cardiovascular and renal comorbidities prior to transplantation [6]. The persistence and progression of CKD after transplant likely reflect the ongoing metabolic risk profile rather than transplant-related factors alone [16]. This underscores the importance of long-term nephrological surveillance and aggressive management of metabolic risk factors in this population.

Interestingly, sepsis was more frequent in the NASH cohort, both in risk and survival analyses. This may be related to the higher prevalence of diabetes mellitus and obesity in NASH patients, both of which are known to impair immune function and increase susceptibility to infections [17]. Additionally, obesity-related alterations in pharmacokinetics of immunosuppressive agents may contribute to either under- or over-immunosuppression, further influencing infection risk [18]. This finding suggests that infectious risk stratification should be particularly emphasized in NASH transplant recipients, especially in the early post-transplant period.

Acute kidney injury was significantly more common among alcoholic transplant recipients. This may be related to perioperative hemodynamic instability, higher prevalence of malnutrition, and potential nephrotoxic exposures in this group. Alcohol-related liver disease patients often present with more severe acute illness at the time of transplantation, which may predispose them to early renal dysfunction [19]. The observed difference highlights the need for careful perioperative renal protection strategies in this subgroup.

A particularly important finding of this study is the absence of a significant difference in liver transplant failure between alcoholic and NASH recipients despite variations in several post-transplant complications. From a transplant systems perspective, this suggests that long-term graft survival remains broadly comparable across both etiologies when contemporary selection criteria and post-transplant care are applied. This observation is clinically reassuring for transplant teams, as it indicates that although morbidity profiles differ, neither indication appears intrinsically disadvantaged with respect to overall graft durability. Additionally, the higher incidence of AKI observed in the alcoholic cohort likely reflects a multifactorial process involving perioperative hemodynamic instability, pre-transplant hepatorenal syndrome physiology, fluctuating intravascular volume status, and potential early calcineurin inhibitor nephrotoxicity [20]. These mechanisms may be particularly relevant in alcohol-related liver disease recipients who frequently present with advanced portal hypertension, malnutrition, and critical illness at the time of transplantation. The concept of etiology-tailored post-transplant management may therefore have practical implications. For example, NASH recipients with significant metabolic syndrome may benefit from steroid-sparing immunosuppressive strategies, earlier consideration of mTOR inhibitor-based regimens, and intensive cardiovascular and metabolic monitoring, whereas alcoholic liver disease recipients may derive benefit from structured psychiatric follow-up, relapse prevention programs, medication adherence reinforcement, nutritional rehabilitation, and closer early renal surveillance.

Importantly, no significant differences were observed in liver transplant failure between the two groups. This suggests that despite differences in specific morbidity patterns, overall graft survival and major failure outcomes remain comparable between alcoholic and NASH-related indications when appropriately matched for baseline characteristics. This finding is clinically reassuring and supports the continued use of liver transplantation in both populations with appropriate selection criteria.

All-cause mortality was modestly but significantly higher in the alcoholic cohort. While the absolute difference was small, the consistent directionality across survival analyses suggests a cumulative effect of multiple adverse outcomes, including rejection, encephalopathy, and renal dysfunction. This emphasizes that alcoholic liver disease remains a high-risk indication even after transplantation and requires comprehensive multidisciplinary long-term care.

For this study, we do note that we utilized the term “nonalcoholic steatohepatitis (NASH)” to define the comparator cohort, consistent with the diagnostic coding practices prevalent in the TriNetX database during the study period. Although the international hepatology community has recently adopted the new nomenclature of metabolic dysfunction-associated steatotic liver disease (MASLD) and metabolic dysfunction-associated steatohepatitis (MASH), our analysis was based on historical ICD-10-CM codes corresponding to the previous definition. Large-scale research has demonstrated a very high degree of concordance (approximately 99%) between patients previously classified as having NASH and those meeting the current MASH criteria, primarily because the vast majority of historical NASH cases already fulfilled the cardiometabolic risk factor requirements of the new definition. Consequently, we believe the post-transplant outcomes observed in our NASH cohort are largely generalizable to contemporary patients with MASH [21].

The strengths of this study include its large sample size, multicenter design, and use of propensity score matching to minimize confounding. However, several limitations must be acknowledged. Residual confounding may persist due to unmeasured variables such as alcohol relapse, immunosuppressive adherence, donor characteristics, and severity of metabolic syndrome. Additionally, reliance on diagnostic coding may introduce misclassification bias. The observational nature of the study also precludes causal inference.

Hence, this study demonstrates that while overall graft failure rates are similar, alcoholic and NASH liver transplant recipients exhibit distinct post-transplant morbidity profiles. These differences highlight the importance of etiology-specific post-transplant management strategies, including enhanced rejection surveillance in alcoholic recipients and aggressive metabolic and infectious risk management in NASH recipients.

## 5. Conclusions

In this large multicenter propensity score-matched cohort study utilizing real-world data from 4601 matched pairs of liver transplant recipients, patients with alcohol-related liver disease demonstrated higher risks of liver transplant rejection, hepatic encephalopathy, acute kidney injury, and all-cause mortality compared with those transplanted for non-alcoholic steatohepatitis (NASH). In contrast, NASH recipients exhibited higher rates of sepsis and chronic kidney disease, while risks of graft failure and ascites were comparable between the two groups. These findings suggest that the underlying etiology of end-stage liver disease may be associated with distinct patterns of post-transplant morbidity and mortality despite balancing measured baseline characteristics. However, the results should be interpreted cautiously given the inherent limitations of retrospective observational analyses based on administrative electronic health record data, including the potential for residual confounding, coding inaccuracies, lack of granular clinical variables, and uncertain generalizability beyond the TriNetX network. Further prospective studies are warranted to validate these findings and to better define etiology-specific post-transplant management strategies that may improve long-term outcomes in both populations.

## Figures and Tables

**Table 1:** Characteristics of cohorts before propensity matching.

Cohort 1 (N = 6876) and Cohort 2 (N = 6471) Characteristics Before Propensity Score Matching							
Demographics							
Cohort			Mean ± SD	Patients	% of Cohort	*p*-Value	Std Diff.
1 2	Age	Current Age	59.0 ± 12.4 63.5 ± 13.5	6781 6432	100% 100%	<0.001	0.344
1 2	AI	Age at Index	53.1 ± 11.1 57.5 ± 13.1	6781 6432	100% 100%	<0.001	0.362
1 2	F	Female		1922 3300	28.3% 51.3%	<0.001	0.483
1 2	2054-5	Black or African American		317 329	4.7% 5.1%	0.241	0.020
1 2	M	Male		4859 3131	71.7% 48.7%	<0.001	0.483
1 2	2106-3	White		5147 5200	75.9% 80.8%	<0.001	0.120
1 2	1002-5	American Indian or Alaska Native		70 43	1.0% 0.7%	0.023	0.040
1 2	UNK	Unknown Race		806 426	11.9% 6.6%	<0.001	0.182
1 2	2076-8	Native Hawaiian or Other Pacific Islander		13 16	0.2% 0.2%	0.484	0.012
1 2	UN	Unknown Ethnicity		1488 1077	21.9% 16.7%	<0.001	0.132
1 2	2186-5	Not Hispanic or Latino		4626 4648	68.2% 72.3%	<0.001	0.089
1 2	2135-2	Hispanic or Latino		667 707	9.8% 11.0%	0.030	0.038
1 2	2131-1	Other Race		324 271	4.8% 4.2%	0.118	0.027
1 2	2028-9	Asian		104 147	1.5% 2.3%	0.002	0.055
Diagnosis							
Cohort			Mean ± SD	Patients	% of Cohort	*p*-Value	Std diff.
1 2	K00-K95	Diseases of the digestive system		5833 5715	86.0% 88.9%	<0.001	0.086
1 2	I00-I99	Diseases of the circulatory system		4634 5142	68.3% 79.9%	<0.001	0.267
1 2	E00-E89	Endocrine, nutritional and metabolic diseases		4522 5326	66.7% 82.8%	<0.001	0.378
1 2	J00-J99	Diseases of the respiratory system		3014 3586	44.4% 55.8%	<0.001	0.228
Medication							
Cohort			Mean ± SD	Patients	% of Cohort	*p*-Value	Std diff.
1 2	CV000	CARDIOVASCULAR MEDICATIONS		4883 4828	72.0% 75.1%	<0.001	0.069
1 2	GA000	GASTROINTESTINAL MEDICATIONS		4819 4701	71.1% 73.1%	0.010	0.045

Cohort 1: Alcohol-related liver disease cohort. Cohort 2: Non-alcoholic steatohepatitis cohort.

**Table 2:** Characteristics of cohorts after propensity matching.

Cohort 1 (N = 4601) and Cohort 2 (N = 4601) Characteristics After Propensity Score Matching							
Demographics							
Cohort			Mean ± SD	Patients	% of Cohort	*p*-Value	Std Diff.
1 2	Age	Current Age	61.1 ± 12.0 61.3 ± 14.2	4601 4601	100% 100%	0.626	0.010
1 2	AI	Age at Index	55.0 ± 10.7 55.3 ± 13.9	4601 4601	100% 100%	0.412	0.017
1 2	F	Female		1722 1679	37.4% 36.5%	0.353	0.019
1 2	2054-5	Black or African American		238 248	5.2% 5.4%	0.641	0.010
1 2	M	Male		2879 2922	62.6% 63.5%	0.353	0.019
1 2	2106-3	White		3651 3660	79.4% 79.5%	0.816	0.005
1 2	1002-5	American Indian or Alaska Native		33 38	0.7% 0.8%	0.551	0.012
1 2	UNK	Unknown Race		379 374	8.2% 8.1%	0.849	0.004
1 2	2076-8	Native Hawaiian or Other Pacific Islander		10 10	0.2% 0.2%	1	<0.001
1 2	UN	Unknown Ethnicity		894 879	19.4% 19.1%	0.692	0.008
1 2	2186-5	Not Hispanic or Latino		3223 3291	70.0% 71.5%	0.119	0.033
1 2	2135-2	Hispanic or Latino		484 431	10.5% 9.4%	0.065	0.039
1 2	2131-1	Other Race		208 190	4.5% 4.1%	0.356	0.019
1 2	2028-9	Asian		82 81	1.8% 1.8%	0.937	0.002
Diagnosis							
Cohort			Mean ± SD	Patients	% of Cohort	*p*-Value	Std diff.
1 2	K00-K95	Diseases of the digestive system		4055 4031	88.1% 87.6%	0.443	0.016
1 2	I00-I99	Diseases of the circulatory system		3493 3476	75.9% 75.5%	0.679	0.009
1 2	E00-E89	Endocrine, nutritional and metabolic diseases		3531 3567	76.7% 77.5%	0.372	0.019
1 2	J00-J99	Diseases of the respiratory system		2356 2332	51.2% 50.7%	0.617	0.010
Medication							
Cohort			Mean ± SD	Patients	% of Cohort	*p*-Value	Std diff.
1 2	CV000	CARDIOVASCULAR MEDICATIONS		3437 3438	74.7% 74.7%	0.981	0.001
1 2	GA000	GASTROINTESTINAL MEDICATIONS		3380 3373	73.5% 73.3%	0.869	0.003

Cohort 1: Alcohol-related liver disease cohort. Cohort 2: Non-alcoholic steatohepatitis cohort.

## Data Availability

The data presented in this study are openly available in TrinetX at https://trinetx.com/solutions/real-world-datasets/ (accessed on 1 April 2026).
